# Chionosphaera pinicorticola sp. nov., a novel basidiomycetous yeast species isolated from pine tree bark in Gyeongju, South Korea

**DOI:** 10.1099/ijsem.0.006622

**Published:** 2025-01-03

**Authors:** Sangsu Lee, Chorong Ahn, Ye-Jin Kim, Seong-Min Choi, Jung-Woo Ko, Changmu Kim, Cheon-Seok Park

**Affiliations:** 1 Department of Food Science and Biotechnology, Kyung Hee University, Yongin 17104, Republic of Korea; 2 Species Diversity Research Division, National Institute of Biological Resources, Incheon 22689, Republic of Korea

**Keywords:** basidiomycetous yeast, *Chionosphaera*, *Chionosphaera cuniculicola*, *Chionosphaera pinicorticola*, phylogenetics, pine tree, *Pinicorticola*

## Abstract

A novel yeast species, isolated from the bark of pine trees in Gyeongju, South Korea, and designated as KCTC 37304^T^ (ex-type KACC 410729), is characterized by its genetic, morphological and physiological properties. Molecular phylogenetic analysis involving the D1/D2 domain of the 26S LSU rRNA gene and the internal transcribed spacer (ITS) region confirms that it belongs to the genus *Chionosphaera*. In comparison to *Chionosphaera cuniculicola* CBS:10065, the type strain of its closest relative, KCTC 37304^T^ exhibits 8 nucleotide substitutions (~2.07%) in the D1/D2 domain and 54 substitutions (~8.47%) in the ITS region. Morphologically, cells of KCTC 37304^T^ are globose to ovoid, which distinguishes them from the cylindrical cells of *C. cuniculicola*. Physiologically, KCTC 37304^T^ does not assimilate methanol or ethanol but can utilize succinate and xylitol, further differentiating it from *C. cuniculicola*. Based on these distinctive genetic, morphological and physiological features, we propose the new species *Chionosphaera pinicorticola* sp. nov., with KCTC 37304^T^ (ex-type strain KACC 410729) designated as the holotype.

## Introduction

*Chionosphaera* (*Chionosphaeraceae*, *Agaricostilbomycetes* and *Pucciniomycotina*), a basidiomycetous genus first described by Cox in 1976, encompasses species primarily associated with tree environments [1]. Wang *et al.* [2] recognized six species within this genus: *Chionosphaera apobasidialis*, *C. coppinsii*, *C. cuniculicola*, *C. erythrinae*, *C. lichenicola* and *C. phylaciicola*. A subsequent molecular re-evaluation resulted in the reclassification of *C. coppinsii* and *C. lichenicola* into a newly established genus, *Crittendenia*, within the *Pucciniomycotina*, thereby positioning them outside the *Chionosphaeraceae* [3].

Historically, these species have been isolated from arboreal habitats – *C. apobasidialis* from the decayed bark of *Quercus macrocarpa*, *C. cuniculicola* from beetles in coniferous forests and *C. erythrinae* from the leaves of *Erythrina tomentosa* – highlighting their ecological preference for tree-associated habitats [4,6]. *Chionosphaera*, when cultured on yeast extract of malt agar, exhibits an ellipsoidal to sausage-shaped morphology, in singles or in pairs. *Chionosphaera* species produce small, white, club-shaped structures known as synnemata on the bark of dead branches of trees [4]. These structures are formed with hyphae, and they are similarly found in other basidiomycetes. Physiologically, *Chionosphaera* species are characterized by their inability to ferment sugars and assimilate nitrates, generally showing a positive reaction to diazonium blue B (DBB) and exhibiting minimal or no urease activity [1].

In our investigation of yeast diversity in Korea, we focused on strains isolated from Gyeongju, where we identified 15 yeast species, including *Aureobasidium* sp., *Curvibasidium* sp., *Filobasidium* sp., *Hannaella* sp., *Kabatiella* sp., *Kwoniella* sp., *Lachancea* sp., *Papiliotrema* sp., *Puccinia* sp., *Rhodotorula* sp., *Schwanniomyces* sp., *Sporisorium* sp., *Torulaspora* sp., *Wickerhamomyces* sp. and *Chionosphaera* sp. across seven native plant species. Among these, a single strain (*Chionosphaera* sp.) was uniquely associated with pine trees. Comprehensive identification of this strain revealed distinct genetic and biochemical characteristics that set it apart from previously described strains. We performed an in-depth analysis of its morphological, physiological and phylogenetic characteristics, leading us to propose a novel species, *Chionosphaera pinicorticola* sp. nov. KCTC 37304^T^. This study contributes to advancing the ecological and biological understanding of this notable genus.

## Methods

### Yeast isolation and identification

*C. pinicorticola* sp. nov. was isolated in June 2023 from the bark of a pine tree located near the historic site Cheomseongdae in Inwang-dong, Gyeongju, Gyeongsangbuk-do (N 35° 50′ 05″ E 129° 13′ 09″), South Korea. This region is characterized by a temperate climate with an average annual temperature of 13.4℃ and average annual precipitation of 1121 mm, based on data collected from 1991 to 2020 (https://data.kma.go.kr/). Pine trees, indigenous to Korea, are prevalent throughout the area. Samples were collected in sterile 50 ml tubes and transported to the laboratory within 3 days. The bark was pulverized using a sterilized mortar and pestle and subsequently macerated overnight in 10 ml of saline solution at pH 3.3 to prepare a suspension [7]. All procedures were performed in sterile conditions to prevent contamination. This suspension was serially diluted to a 10^−6^ concentration, and 200 µl aliquots were plated onto yeast extract-peptone dextrose (YPD) agar supplemented with 100 µg ml^−1^ ampicillin and dichloran rose bengal chloramphenicol agar. Incubation was carried out at 25℃ for 3 days [8]. Emerging yeast colonies were isolated, subcultured onto fresh YPD medium and incubated under identical conditions [9]. Purified strains were preserved in 25% glycerol at −80 °C for long-term storage [10]. Genomic DNA extraction was performed with slight modification as described in the previous study [11]. Yeast cells were collected by centrifugation at 13,000 ***g*** for 5 min. Liquid nitrogen was employed to disrupt the cell wall, followed by the addition of 500 µl of lysis buffer (200 mM Tris-HCl, pH 8.5; 250 mM NaCl; 25 mM EDTA; and 0.5% SDS) and 500 µl of phenol/chloroform/isoamyl alcohol (25 : 24 : 1). The cell lysate was then centrifuged at 13,000 ***g*** for 30 min to pellet debris. To the supernatant, 1/10 vol of 3 M sodium acetate (50 µl for every 500 µl of supernatant) and 2.5 volumes of isopropyl alcohol were added to precipitate the genomic DNA. The DNA was subsequently purified using an ethanol precipitation method (adding 2.5 volumes of 100% ethanol), followed by resuspension in 100–200 µl of TE buffer containing RNase.

### DNA sequencing and phylogenetic analysis

DNA sequencing targeted the D1/D2 domain of the 26S LSU rRNA gene and the internal transcribed spacer (ITS) region [12,13]. Primers NL-1 (GCATATCAATAAGCGGAGGAAAAG) and NL-4 (GGTCCGTGTTTCAAGACGG) were employed for amplifying the D1/D2 domain, while ITS-1 (TCCGTAGGTGAACCTGCGG) and ITS-4 (TCCTCCGCTTATTGATATGC) were used for the ITS region. The PCR protocol included an initial denaturation at 95 °C for 5 min, followed by 30 cycles of denaturation at 95 °C for 30 s, annealing at 55 °C for 30 s and extension at 72 °C for 30 s, concluding with a final extension at 72 °C for 10 min. PCR amplification was carried out using Ex *Taq* polymerase (Takara, Shiga-ken, Japan). Sequences were aligned and assembled using SeqMan software and compared against the GenBank database via the BLASTn search tool [14,15]. Phylogenetic relationships were elucidated using mega X software, employing the maximum-likelihood (ML) methods based on the Tamura-Nei model, with bootstrap analysis from 1000 replicates confirming the robustness of the clades [16,17]. To confirm the taxonomic relationships of the isolates, the sequences of members of the basidiomycetous yeast genus were obtained from NCBI, and a phylogenetic tree was constructed as reported in previous studies [18]. The general time reversible models were used for the ML analyses [19].

### Physiological and morphological characterization

The strains underwent physiological characterization employing conventional methodologies as described by Kurtzman *et al.* [20]. Assays included carbon and nitrogen compound assimilations, urea hydrolysis, acetic acid tolerance, high osmotic pressure resilience, DBB colouration test and extracellular amyloid compound formation test (indicative of starch synthesis). All assays were conducted on agar plates over a period of 7 days at 25 °C and replicated three times to ensure reliability. Morphological observations were facilitated by culturing cells on YM agar for 3 days at 25 °C, followed by microscopic examination using a DN-10A light microscope (Samwon, Goyang, Korea). Colony characteristics and hyphal formations were observed after culturing on yeast malt (YM) agar for 7 days and on Dalmau plates with cornmeal agar for 14 days, respectively. Cells harvested directly from agar plates were analysed immediately without undergoing washing or fixation procedures. Imaging was performed using ScopeImage 9.0 software (Guangzhou Micro-shot Technology Co., Ltd., MSHOT). Subsequently, cellular morphology was meticulously examined under a DN-10A light microscope (Samwon).

## Results and discussion

### Novel species identification and delineation

In our investigation of yeast diversity in Korea, we identified 19 yeast species from Gyeongju. These strains were isolated from various ecological niches. Notably, one strain exhibited exclusive association with pine trees. Comprehensive analysis of this strain revealed distinctive genetic characteristics that are akin to the genus *Chionosphaera* yet sufficiently differentiate it from established *Chionosphaera* species, including *C. apobasidialis*, *C. coppinsii*, *C. cuniculicola*, *C. lichenicola* and *C. phylacicola*.

A comprehensive review of the genus *Chionosphaera*, its species and their ecological and physiological attributes is well documented by Kwon-Chung [1]. This genus, categorized within the *Agaricostilbales* order of the *Agaricostilbomycetes* class, under the *Pucciniomycotina* subphylum, encompasses several species, including *C. apobasidialis*, *C. coppinsii*, *C. cuniculicola*, * C. lichenicola* and *C. phylacicola* [21]. These species predominantly inhabit tree bark and other natural substrates. Similar to other members of this genus, the newly identified species KCTC 37304^T^ was isolated from the bark of a pine tree. Although the full spectrum of ecological traits for this species *in situ* remains to be elucidated, its detection in analogous arboreal environments implies that its ecological preferences are consistent with those of its congeners.

**Fig. 1.**
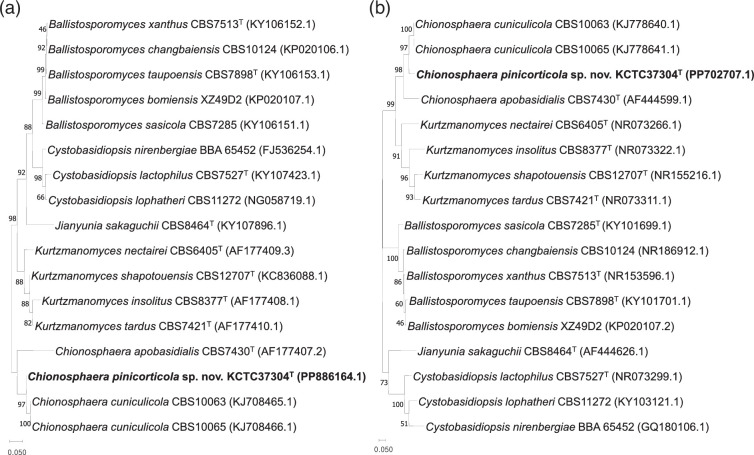
Phylogenetic tree based on the sequences of the 26S rRNA gene D1/D2 domain (**a**) and the internal transcribed spacer (ITS) region (**b**), showing positions of *Chionosphaera pinicorticola* sp. nov. KCTC 37304^T^ with respect to closely related species. Numbers at the node indicate percentages of bootstrap value of maximum-likelihood analyses, derived from 1000 samples. Bar, 0.05 substitutions per site in the D1/D2 domain of the LSU rRNA gene and the ITS region.

Phylogenetic analyses of KCTC 37304^T^, utilizing the D1/D2 domain sequences of the 26S LSU rRNA gene and the ITS region, corroborated its inclusion within the *Chionosphaera* genus, showing a close relationship to *C. cuniculicola* CBS:10065. Notable genetic divergence was observed; KCTC 37304^T^ displayed approximately 2.07% divergence (eight nucleotide substitutions) in the D1/D2 domain and 8.47% divergence (54 substitutions) in the ITS region relative to *C. cuniculicola* CBS:10065. Phylogenetic reconstructions positioned KCTC 37304^T^ within a distinct subclade of the *Chionosphaera* clade, supported by robust bootstrap values (Fig. 1). According to Kurtzman and Robnett, strains with more than 1% nucleotide substitutions in the approximately 600-bp D1/D2 domain may be classified as separate species [13]. KCTC 37304^T^ strain differs significantly from previously described species within the clade, indicating a degree of sequence divergence that may support its classification as a separate species.

Table 1 shows the physiological characteristics of KCTC 37304^T^ regarding carbon source assimilations, in comparison to its closest relative, *C. cuniculicola* [4]. Unlike *C. cuniculicola*, KCTC 37304^T^ is unable to assimilate methanol and ethanol but can utilize succinate and xylitol (Table 1). Morphologically, while *C. cuniculicola* typically exhibits fusiform to allantoid cells, KCTC 37304^T^ displays a range of globose to ovoid and ellipsoidal cell forms, often occurring singly or in pairs with polar budding. Based on these distinctive genetic, physiological and phenotypic characteristics, we propose recognizing KCTC 37304^T^ as a new species, *C. pinicorticola* sp. nov. KCTC 37304^T^.

**Table 1.** Physiological characterization (carbon assimilation sources) of *Chionosphaera pinicorticola* sp. nov. KCTC 37304^T^ compared to its closest relative, *C. cuniculicola* 1, *C. pinicorticola* sp. nov. KCTC 37304^T^ incubated for a week; 2, *C. pinicorticola* sp. nov. KCTC 37304^T^ incubated for 3 weeks; 3, the carbon assimilation characteristics of *C. cuniculicola* in the previous study [4]; +, positive; −, negative; w, weak positive; s, slow; v, variable; nd: not detected).

Carbon source	1	2	3	Carbon source	1	2	3
ᴅ-Glucose	+	+	+	ᴅ-Galactose	−	w	w
ʟ-Sorbose	−	w	+	ᴅ-Glucosamine	−	−	−
ᴅ-Ribose	−	−	−	ᴅ-Xylose	−	−	−
ʟ-Arabinose	−	w	s	ᴅ-Arabinose	w	w	+
ʟ-Rhamnose	−	−	−	Sucrose	−	−	−
Maltose	−	−	−	Trehalose	+	+	v
*α*-Methyl-ᴅ-glucoside	−	−	−	Cellobiose	w	w	−
Salicin	−	−	v	Arbutin	−	−	nd
Melibiose	−	−	s/w	Lactose	−	−	−
Raffinose	−	−	−	Melezitose	w	w	−
Inulin	+	+	s	Soluble starch	w	+	v
Glycerol	+	+	+	Erythritol	−		−
Ribitol	w	+	v	Xylitol	w	+	−
ʟ-Arabinitol	w	+		ᴅ-Glucitol	+	+	s
ᴅ-Mannitol	+	+	+	Galactitol	−	−	−
Myo-Inositol	−	−	s/w	ᴅ-Glucono-1,5-lactone	−	−	nd
2-Keto- ᴅ-gluconate	−	−	nd	5-Keto-ᴅ-gluconate	−	−	nd
ᴅ-Gluconate	w	w	nd	ᴅ-Glucuronate	−	−	nd
ᴅ-Galacturonic acid	−	−	nd	ᴅʟ-Lactate	−	w	−
Succinate	w	+	−	Citrate	+	+	−
Methanol	−	−	+	Ethanol	−	−	+
Propane 1,2-diol	+	+	nd	Butane 2,3-diol	w	w	nd
Quinic acid	−	−	nd	ᴅ-Glucarate	−	−	nd

### Description of *Chionosphaera pinicorticola* sp. nov. KCTC 37304^T^

*Chionosphaera pinicorticola* [pi.ni.cor.ti’co.la. L. fem. n. *pinus*, pine tree; L. masc./fem. n. *cortex*, bark; L. suff. *–cola* (from L. masc./fem. n. *incola*), inhabitant; N.L. fem. n*. pinicorticola*, inhabitant of bark]

**Fig. 2.**
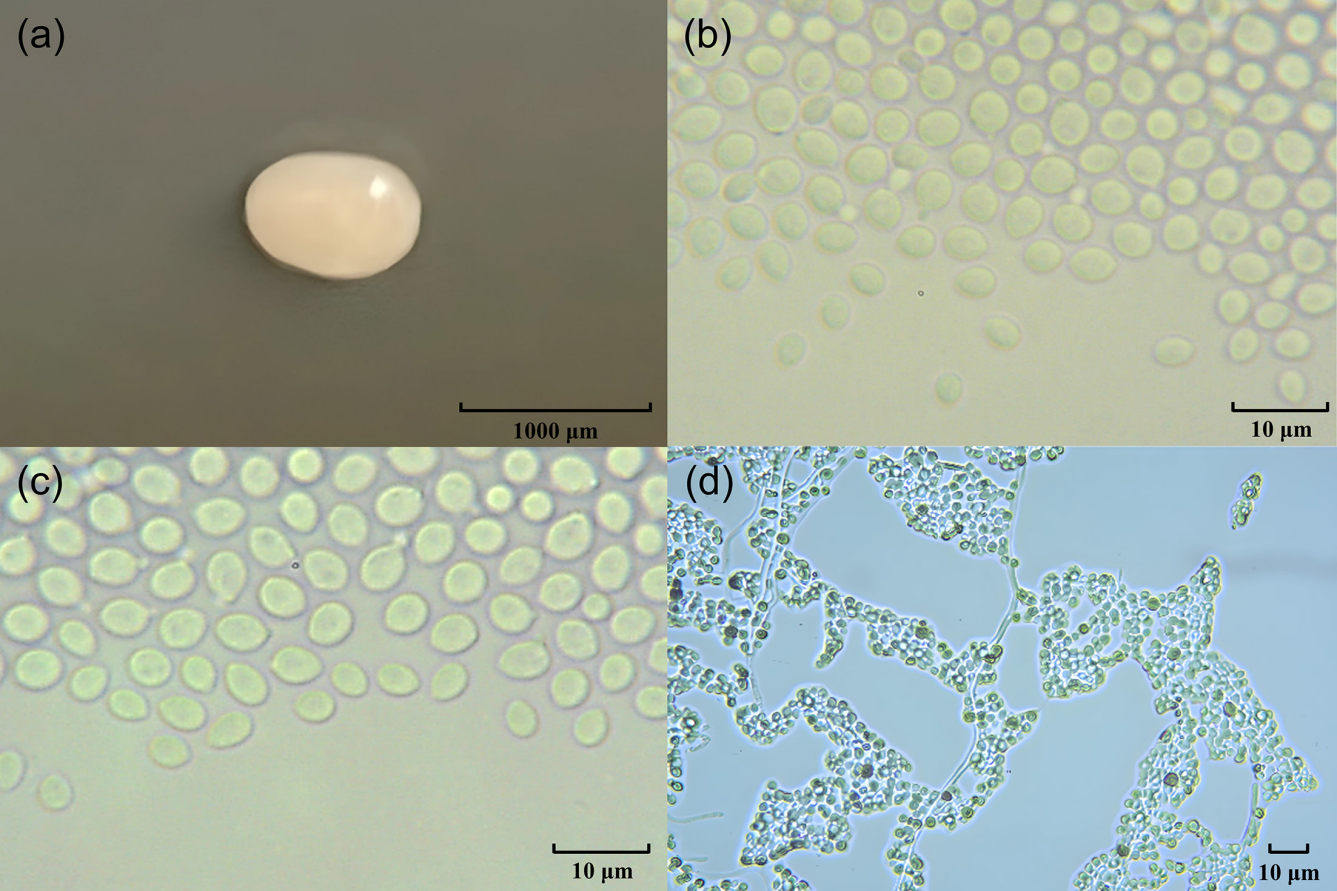
Morphology of *C. pinicorticola* sp. nov. KCTC 37304^T^ colony on yeast malt (YM) agar after 7 days at 25 °C (**a**). Budding cells on YM agar after 3 days at 25 °C (**b**) and after 7 days at 25 °C (**c**). Pseudohyphal formation on Dalmau plates containing cornmeal agar after 2 weeks (**d**).

*C. pinicorticola* sp. nov. produces white to cream-coloured round-shaped colonies on YM agar with an entire margin (Fig. 2). The cells are globose, ovoid to ellipsoidal, and occur singly or in pairs with polar budding [20]. After culturing the strain for 2 weeks in a Dalmau plate, however, hyphae were observed, suggesting that they may have the filamentous stage. Similar to * C. cuniculicola*, this species abundantly produces hyaline hyphae without clamp connections after 3 days on cornmeal agar. Basidial structures were not observed in *C. pinicorticola* sp. nov. KCTC 37304^T^ during this study. However, the possibility of the basidial structures development, not yet observed, may not be eliminated because *C. apobasidialis* and *C. cuniculicola* do not usually develop basidial structures, unless there are dematiaceous fungi such as *Cladosporium* spp. present as a contaminant in the culture [1,4]. The following carbon sources are assimilated: ᴅ-glucose, ᴅ-galactose (weak), ʟ-sorbose (weak), ʟ-arabinose (weak), ᴅ-arabinose (weak), trehalose, cellobiose (weak), melezitose, inulin, soluble starch, glycerol, ribitol, xylitol, ʟ-arabinitol, ᴅ-glucitol, ᴅ-mannitol, ᴅ-gluconate, ᴅl-lactate (weak), succinate, citrate, propane 1,2-diol and butane 2,3-diol (weak). ᴅ-Glucosamine, ᴅ-ribose, ᴅ-xylose, ʟ-rhamnose, sucrose, maltose, α-methyl-ᴅ-glucoside, salicin, arbutin, melibiose, lactose, raffinose, erythritol, galactitol, myo-inositol, ᴅ-glucono-1,5-lactone, 2-keto-ᴅ-gluconate, 5-keto-ᴅ-gluconate, ᴅ-glucuronate, ᴅ-galacturonic acid, succinate, methanol, ethanol, quinic acid and ᴅ-glucarate are not assimilated as carbon sources (Table 1). Unlike *C. cuniculicola*, *C. pinicorticola* sp. nov. is unable to assimilate methanol and ethanol, but it can utilize succinate and xylitol. Ethylamine, ʟ-lysine, cadaverine, ᴅ-glucosamine and ᴅ-tryptophan are assimilated as nitrogen sources. Potassium nitrate, sodium nitrate, creatine, creatinine and imidazole are also assimilated as nitrogen sources (Table S1, available in the online Supplementary Material’). * C. pinicorticola* does not grow at high osmotic pressures such as 50% glucose agar, 60% glucose agar, 10% NaCl agar and 16% NaCl agar (Table S2). There is no hydrolysis of urea and no starch formation. The DBB reaction is positive.

Strain KCTC 37304^T^ is designated as the holotype of *C. pinicorticola* sp. nov. The isolate was collected in June 2023 from the bark of a pine tree in Inwang-dong, Gyeongju, Gyeongsangbuk-do, South Korea. The holotyope is permanently preserved as metabolically inactive culture (−80 °C) at the Korean Collection for Type Cultures (KCTC) as KCTC 37304^T^. The ex-type culture has been preserved in the Korean Agricultural Culture Collection (KACC) as KACC 410729. Both the sequences of the D1/D2 domain of the 26S rRNA gene (PP702707) and the ITS region (PP886164) of this strain have been deposited in NCBI GenBank.

## Supplementary material

10.1099/ijsem.0.006622 Uncited Table S1.

## Abbreviations

DBB: diazonium blue B
ITS: internal transcribed spacer
KACC: Korean Agricultural Culture Collection
KCTC: Korean Collection for Type Cultures
ML: maximum-likelihood
YM: yeast malt
YPD: yeast extract-peptone dextrose
