# Supplementary material for: Chionosphaera pinicorticola sp. nov., a novel basidiomycetous yeast species isolated from pine tree bark in Gyeongju, South Korea
Source: Int J Syst Evol Microbiol. 2025 Jan 3;75(1):006622. doi: 10.1099/ijsem.0.006622 (PMC12282024; doi:10.1099/ijsem.0.006622)
Supplement: Uncited Table S1. [file ijsem-75-06622-s001.pdf]

Supplementary Tables

Table S1. Physiological characteristics of *C. pinicorticola* sp. nov. KCTC 37304^T about assimilation of nitrogen source. 1, *C. pinicorticola* sp. nov. KCTC 37304^T incubated for 3 days.; 2, *C. pinicorticola* sp. nov. KCTC 37304^T incubated for 1 week; 3, the incubation of *C. pinicorticola* sp. nov. KCTC 37304^T for 2 weeks; +, Positive; –, negative; w, weak positive.

Nitrogen sources	1	2	3
Potassium nitrate	-	-	-
Sodium nitrite	-	-	-
Ethylamine	+	+	+
L-Lysine	+	+	+
Cadaverine	+	+	+
Creatine	-	-	-
Creatinine	-	-	-
D-Glucosamine	+	+	+
Imidazole	-	-	-
D-Tryptophan	w	w	w
Ammonium sulfate	+	+	+

Table S2. Physiological characteristics of *C. pinicorticola* sp. nov. KCTC 37304^T about test for tolerance of 1% of acetic acid and test for high osmotic pressure. 1, *C. pinicorticola* sp. nov. KCTC 37304^T incubated for a week; 2, *C. pinicorticola* sp. nov. KCTC 37304^T incubated for 3 weeks; -: no sign of colony; +: well-formed colony on the plate

Culture media	1	2
50% Glucose agar	-	-
60% Glucose agar	-	-
10% NaCl agar	+	+
16% NaCl agar	+	+
1% YE agar	+	+
1% of Acetic acid	-	-
